# High-resolution non-line-of-sight imaging based on liquid crystal planar optical elements

**DOI:** 10.1515/nanoph-2023-0655

**Published:** 2024-01-10

**Authors:** Zhibin Zhao, Qi Zhang, Xiaoyin Li, Yinghui Guo, Mingbo Pu, Fei Zhang, Hengshuo Guo, Zewei Wang, Yulong Fan, Mingfeng Xu, Xiangang Luo

**Affiliations:** National Key Laboratory of Optical Filed Manipulation Science and Technology, Institute of Optics and Electronics, Chinese Academy of Sciences, Chengdu 610209, China; State Key Laboratory of Optical Technologies on Nano-Fabrication and Micro-Engineering, Institute of Optics and Electronics, Chinese Academy of Sciences, Chengdu 610209, China; Research Center on Vector Optical Fields, Institute of Optics and Electronics, Chinese Academy of Sciences, Chengdu 610209, China; School of Optoelectronics, University of Chinese Academy of Sciences, Beijing 100049, China; Tianfu Xinglong Lake Laboratory, Chengdu 610299, China

**Keywords:** metasurface, liquid crystal planar optical element, non-line-of-sight imaging

## Abstract

Non-line-of-sight (NLOS) imaging aims at recovering hidden objects located beyond the traditional line of sight, with potential applications in areas such as security monitoring, search and rescue, and autonomous driving. Conventionally, NLOS imaging requires raster scanning of laser pulses and collecting the reflected photons from a relay wall. High-time-resolution detectors obtain the flight time of photons undergoing multiple scattering for image reconstruction. Expanding the scanning area while maintaining the sampling rate is an effective method to enhance the resolution of NLOS imaging, where an angle magnification system is commonly adopted. Compared to traditional optical components, planar optical elements such as liquid crystal, offer the advantages of high efficiency, lightweight, low cost, and ease of processing. By introducing liquid crystal with angle magnification capabilities into the NLOS imaging system, we successfully designed a large field-of-view high-resolution system for a wide scanning area and high-quality image reconstruction. Furthermore, in order to reduce the long data acquisition time, a sparse scanning method capitalizing on the correlation between measurement data to reduce the number of sampling points is thus proposed. Both the simulation and experiment results demonstrate a >20 % reduction in data acquisition time while maintaining the exact resolution.

## 1 Introduction

Non-line-of-sight imaging (NLOS) aims to reconstruct three-dimensional (3D) objects obscured from the detector’s direct line of sight. This technique has attracted considerable attention due to its promising applications in diverse fields, including autonomous driving, robotic vision, rescue operations, remote sensing, and the medical sector. The current NLOS imaging methods mainly include photon time-of-flight imaging [1]–[12], coherent imaging [[Bibr j_nanoph-2023-0655_ref_013] [Bibr j_nanoph-2023-0655_ref_014] [Bibr j_nanoph-2023-0655_ref_015] [Bibr j_nanoph-2023-0655_ref_016] [Bibr j_nanoph-2023-0655_ref_017], intensity imaging [19], [20], [21], speckle imaging [22], [23], wavefront shaping [17], [24], and deep learning [25], [26], [27]. Photon time-of-flight imaging is the most widely used approach, owing to its exceptional imaging quality and precision. However, its resolution is primarily limited by the scanning area on the relay wall rather than the numerical aperture of the collecting optical system. The scanning area on the relay wall can be considered the virtual aperture of the NLOS imaging system, which determines the amount of detectable photon time-of-flight data. Expanding the scanning area is a crucial way to improve the resolution of NLOS imaging. However, current scanning systems in NLOS imaging are typically limited by maximum scanning angle, preventing further expansion of the scanning area. Introducing a beam steering system composed of traditional optical elements in front of the scanning system is a common method to expand the scanning area, but the bulky size of traditional optical components restricts further applications of NLOS imaging.

Recently, metasurface has received extensive research interest due to its high integration, compact size, and multifunctionality [[Bibr j_nanoph-2023-0655_ref_028] [Bibr j_nanoph-2023-0655_ref_029] [Bibr j_nanoph-2023-0655_ref_030] [Bibr j_nanoph-2023-0655_ref_031]. Metasurface can manipulate the amplitude, phase, and polarization of incident waves by tailoring each meta-atom’s morphology [[Bibr j_nanoph-2023-0655_ref_033] [Bibr j_nanoph-2023-0655_ref_034] [Bibr j_nanoph-2023-0655_ref_035] [Bibr j_nanoph-2023-0655_ref_036] [Bibr j_nanoph-2023-0655_ref_037] [Bibr j_nanoph-2023-0655_ref_038] [Bibr j_nanoph-2023-0655_ref_039] [Bibr j_nanoph-2023-0655_ref_040]. Moreover, it has demonstrated its fast advancement in many fields, such as vortex recognition [42], optical encryption [38], and wavefront shaping [43]. In addition, metasurfaces also perform excellently in controlling diffraction [44] and enhancing photon upconversion [45]. Similar to metasurfaces, which impart abrupt phase shifts to incident waves at the interface, liquid crystal (LC) molecules with spatially varying orientations can also form desired phase profiles by exploiting the Pancharatnam–Berry (PB) geometric phase [[Bibr j_nanoph-2023-0655_ref_046] [Bibr j_nanoph-2023-0655_ref_047] [Bibr j_nanoph-2023-0655_ref_048] [Bibr j_nanoph-2023-0655_ref_049] [Bibr j_nanoph-2023-0655_ref_050] [Bibr j_nanoph-2023-0655_ref_051] [Bibr j_nanoph-2023-0655_ref_052] [Bibr j_nanoph-2023-0655_ref_053] [Bibr j_nanoph-2023-0655_ref_054]. Furthermore, LCs can be electrically controlled to achieve reconfigurability [56]. This characteristic makes LCs an ideal material for various applications across different domains, including display technology [57], optical modulation [58], and antenna design [59]. In contrast to the complex lithographic manufacturing process of metasurfaces, the fabrication of LC planar optical elements (POEs) only involves coating or filling liquid crystal materials onto a substrate for preparation [60]. Combining two LC-POEs to construct a miniature telescope, an efficient, wide-angle, and high-precision beam deflection has been achieved [61]. Inspired by these advancements, it is anticipated that integrating an electrically tunable LC beam steering device into NLOS imaging can result in a larger scanning area.

In this work, we have devised a large scanning range system consisting of a confocal NLOS imaging system and an angle magnification system. By constructing a telescope module with two cascaded LC POEs, we can achieve high optical efficiency, minimal wavefront distortion, and angle magnification independent of the incident spatial position. In the large scanning range system, the peak signal-to-noise ratio (PSNR) of the reconstructed results can be increased by about 5 dB, and the root mean square error (RMSE) can be reduced by approximately 15 %. However, achieving high-resolution reconstruction via the large scanning range system requires the collection of dense grid measurements, significantly elongating the data acquisition time. For further optimization, we proposed a novel sparse scanning method based on spatial correlation to reduce the redundant measurements. This distinctive scanning approach leverages the spatial correlation [62], [63] of measured data between adjacent scanning positions. By comparing the spatial correlation of measurement data from neighboring points across the entire scanning area, we found that the spatial correlation in the central region is higher than that in the edge region. Consequently, sparse scanning can be adopted for the center area of the relay wall. In simulation, our method achieves a 1.5-fold increase in reconstruction resolution while saving up to 26 % of the scanning time. In experiments, our method can increase the PSNR of the reconstructed image by approximately 5 dB and reduce the RMSE of the reconstructed image by 15 % while saving up to 23 % of the scanning time.

## 2 Results and discussion

### 2.1 Impact of scanning area and sampling rate on the lateral resolution

**Figure 1:**
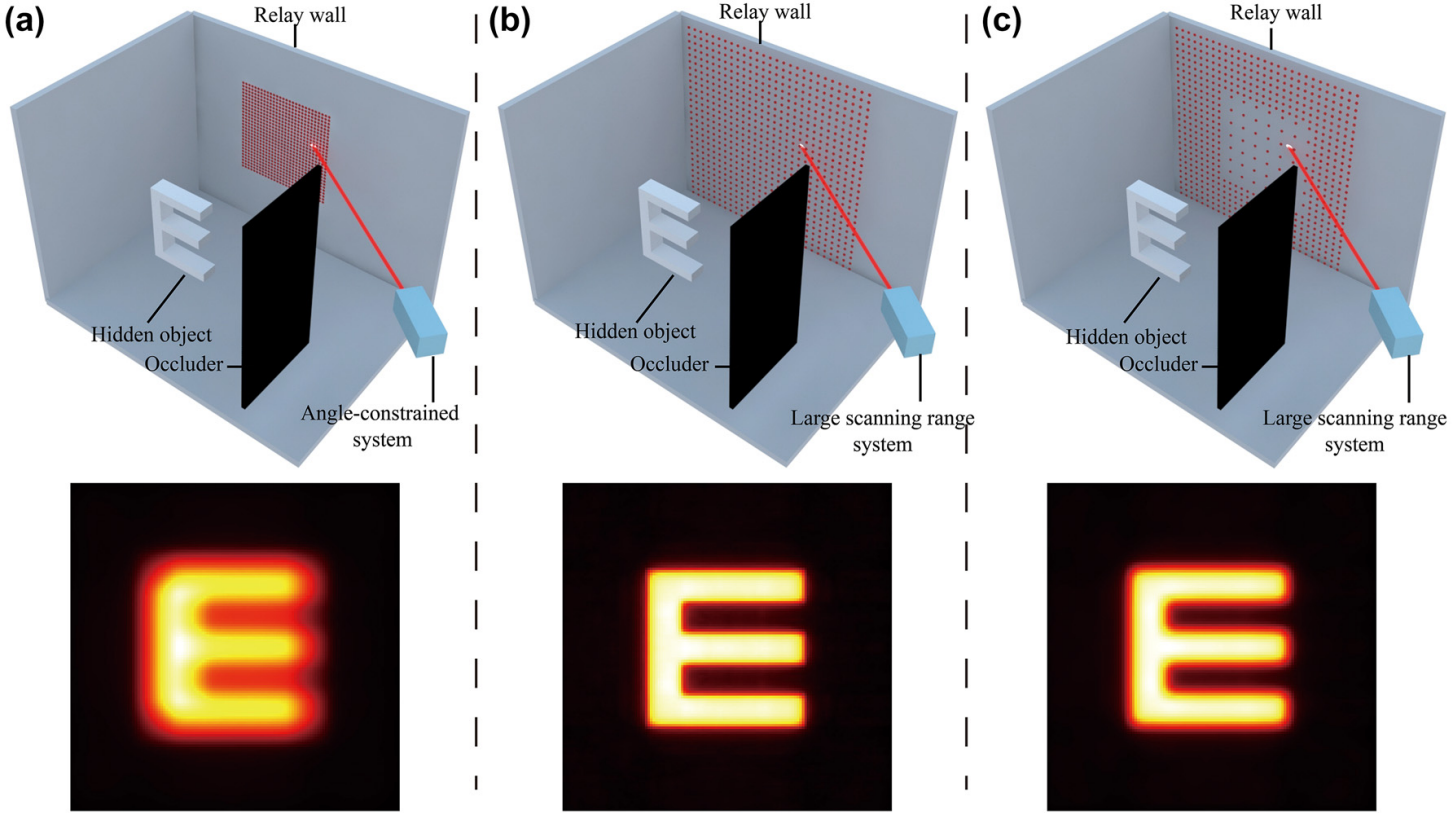
Overview of large scanning range NLOS imaging scene. (a) Angle-constrained NLOS imaging scene. (b) Large scanning range NLOS imaging scene. (c) Sparse scanning NLOS imaging scene.

An angle-constrained NLOS imaging system, as shown in Figure 1a, records the time-of-flight of scattered photons containing information about hidden objects by scanning the relay wall in a dense grid pattern. Through reconstruction algorithms, the shape and position of the hidden objects can be recovered. In contrast to the angle-constrained NLOS system, the large scanning range system scans a broader area on the relay wall, as shown in Figure 1b, which allows the detector to capture a greater amount of scattered light, signifying a more extensive recording of photon time-of-flight containing valuable object information. Consequently, the reconstruction results obtained from the large scanning range system are smoother, with less background noise. Furthermore, in the large scanning range system, a broader scanning area supports the detector to resolve the time-of-flight of more photons, thus achieving higher reconstructed lateral resolution. Specifically, in a non-confocal system, the minimum resolvable spacing Δ*d* is

(1) $$\begin{array}{c} \Delta d\geq \frac{\sqrt{w^{2}+z^{2}}\left(c\Delta t-\delta x\right)}{w} \end{array}$$

where 2*w* is the scanning width or height of the visible wall, *c* is the light speed, *z* is the distance between the hidden object and the relay wall, and *δx* is the path difference of the hidden object from the detection point on the relay wall (see Supplementary Material Note S1 for details). In a non-confocal system, the detector is typically aimed at the center position of the wall, so that *δx* takes a very small value. In contrast, in the confocal system, the minimum resolvable distance can be expressed as:

(2) $$\begin{array}{c} \Delta d\geq \frac{c\Delta t\sqrt{w^{2}+z^{2}}}{2w} \end{array}$$

According to Equations (1) and (2), when the hidden object remains static, the minimum resolution of the reconstructed image is related to both the scanning area and the minimum resolution of the detection system. Therefore, increasing the scanning area is an important way to improve the resolution of the reconstructed image. However, while enlarging the scanning area, the scanning interval also increases. According to the Nyquist sampling theorem, the maximum scanning interval Δ*x* is

(3) $$\begin{array}{c} \Delta x<\frac{\Delta d}{2} \end{array}$$

where Δ*d* represents the minimum distance defined in Equations (1) and (2). Equation (3) indicates that the maximum scanning interval should be less than half of the theoretical minimum reconstruction resolution. In the Supplementary Material Note S2, we specifically discuss the impact on the reconstruction results under different undersampling conditions through simulations.

Equations (1)–(3) show that achieving high lateral resolution in NLOS imaging requires large and dense scanning of the relay wall surface. Each scanning position requires a certain exposure time for the detector to collect measurement data, which significantly reduces the speed of NLOS imaging. To alleviate this problem, we adopt sparse scanning into the large scanning range imaging system to accelerate the scanning speed with high spatial resolution due to the high spatial correlation among the measured positions, as revealed in Figure 1c. The lateral resolution of the reconstructed image from the sparse scanning is slightly lower as compared to the large scanning range system but far better than that of the angle-constrained system. Utilizing sparse scanning in the large scanning range system allows for a significant reduction in scanning time at the cost of sacrificing minimal reconstruction resolution.

### 2.2 Liquid crystal planar optical elements for angle magnification

In the large scanning range system, the POEs are responsible for achieving a larger scanning angle by steering the output beam through tailoring its geometric phase profile. We choose POEs based on LC materials, whose anisotropy allows to alter the alignment direction of LC molecules to create an LC geometric phase. Assume that the local optical axis of the LC molecule has an orientation angle of *φ* with respect to the *x*-axis. The Jones matrix can be represented as:

(4) $$\begin{array}{ll} J & =M\left[\begin{array}{cc} e^{-\frac{i\Gamma }{2}} & 0\\ 0 & e^{\frac{i\Gamma }{2}} \end{array}\right]M^{-1}\\ & =\left[\begin{array}{cc} \cos\varphi & -\sin\varphi \\ \sin\varphi & \cos\varphi \end{array}\right]\left[\begin{array}{cc} e^{-\frac{i\Gamma }{2}} & 0\\ 0 & e^{\frac{i\Gamma }{2}} \end{array}\right]\left[\begin{array}{cc} \cos\varphi & \sin\varphi \\ -\sin\varphi & \cos\varphi \end{array}\right] \end{array}$$

where *M* is the rotation matrix, Γ is the LC phase retardation. For a circularly polarized incident light $E_{i}=\left[\begin{array}{c} 1\\ i\sigma \end{array}\right]$ , the output light can be calculated as:

(5) $$\begin{array}{c} E_{o}=J\cdot E_{i}=\frac{1}{\sqrt{2}}\left[\begin{array}{c} 1\\ i\sigma \end{array}\right]\cos\frac{\Gamma }{2}-\frac{i}{\sqrt{2}}\left[\begin{array}{c} 1\\ -i\sigma \end{array}\right]\sin\frac{\Gamma }{2}e^{i2\sigma \varphi } \end{array}$$

where *σ* = ±1, corresponding to left-circularly polarized and right-circularly polarized light, respectively. The second term on the right side of Equation (5) indicates that the *E*_*i*_ is converted to the orthogonal polarization carrying an additional geometric phase term *e* ^*i*2*σφ*^. The geometric phase term indicates that a continuous phase variation from 0 to 2*π* can be achieved by rotating the LC molecules from 0 to *π*. Furthermore, the efficiency of the output light modulated by the geometric phase is sin^2^(Γ/2).

**Figure 2:**
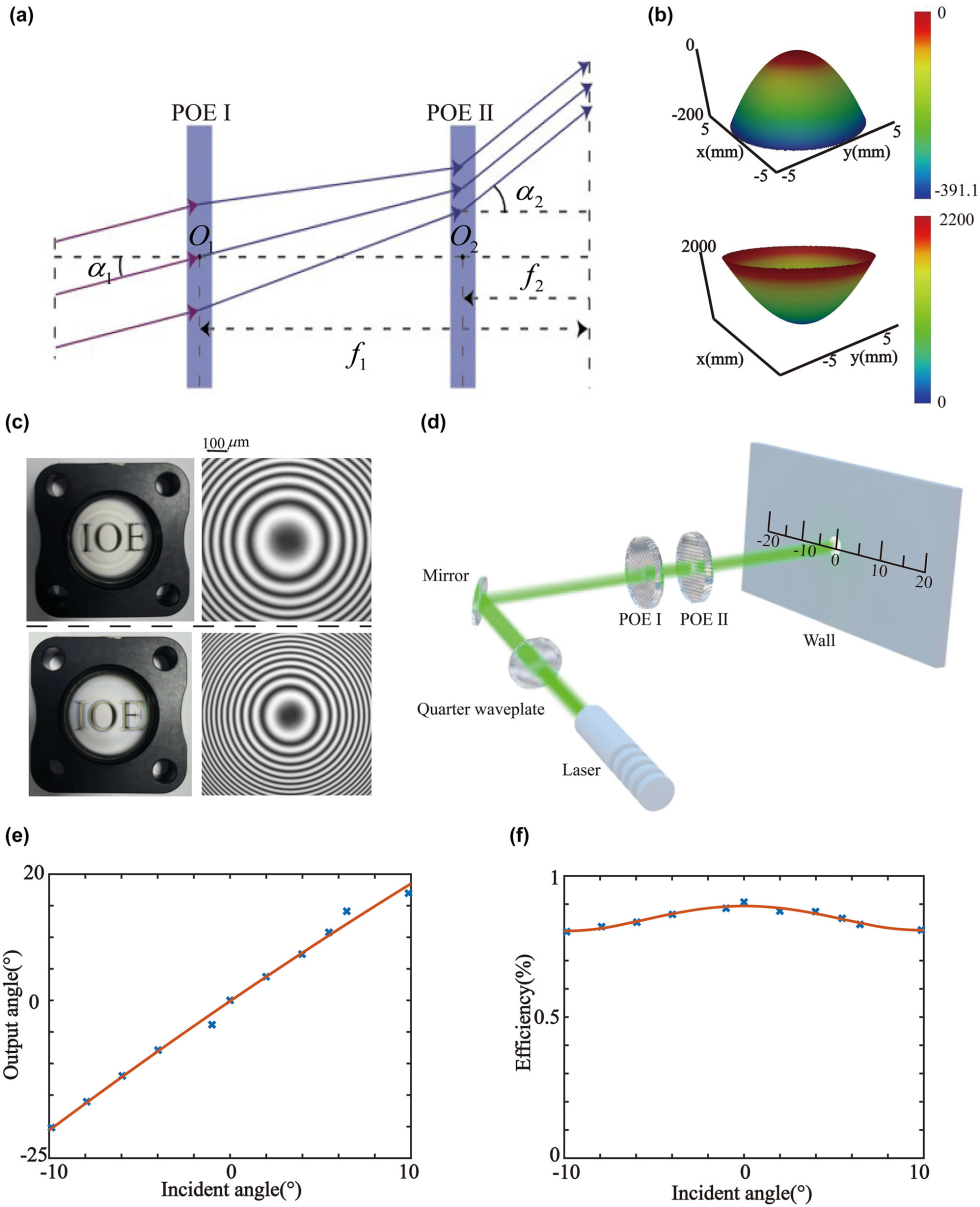
The work principle and test results of the cascaded POEs. (a) Conceptual scheme of the cascaded POE for amplification of beam steering. (b) Phase profiles of the POE I and POE II. The upper image is POE I, and the lower image is POE II. (c) Samples and polarized optical microscopic images of POE I and POE II. The upper image is POE I, and the lower image is POE II. (d) Schematic of the measurement setup. (e) Performance of the deflection angle. (f) Performance of the angular efficiency.

Here, we use these two POEs to develop a planar telescope, as schematically shown in Figure 2a. In the optical system, the POE I acts as an objective to converge the incident beam with a focal length of *f*_1_. Then, the incident beam propagates through POE II, which functions as an eyepiece with a focal length of *f*_2_ and ultimately delivers a collimated beam. When the incident beam with an oblique angle of *θ*_1_ passes through the cascaded POE with an increased deflection angle of *θ*_2_, the magnification factor can then be defined as:

(6) $$\begin{array}{c} M=\frac{\theta_{2}}{\theta_{1}}\approx -\frac{f_{1}}{f_{2}} \end{array}$$

Therefore, the cascaded POE based on the Galileo telescope system is implemented for beam steering. Commercial software (Zemax OpticStudio) is used to optimize the phase profiles. The simulation is performed with a 532 nm monochromatic light source. The distance between the two POEs is 38 mm. The optimized design is obtained by minimizing the size of the output beam while maintaining the correct emission angle. The phase profiles of POEs are obtained as follows:

(7) $$\begin{array}{c} \phi \left(r\right)=\alpha {\left(\frac{r}{R}\right)}^{2}+\beta {\left(\frac{r}{R}\right)}^{4}+\gamma {\left(\frac{r}{R}\right)}^{6} \end{array}$$

where *r* is the radial coordinate, *R* is the radius of POEs (2.895 mm for POE I and 4.90055 mm for POE II), and *α*, *β*, and *γ* are constant coefficients. The optimized phase profiles are shown in Figure 2b. The color bar represents phase values *ϕ*.

The fabricated POEs are shown in Figure 2c, 0.3 mm above the sample. The *f*-numbers of POE I and POE II are 13.1 and 3.9, respectively. The polarized optical microscopic images of the POEs are shown on the right side of each POE. To evaluate the performance of each POE, we construct a measurement setup, as shown in Figure 2d. A 532 nm linearly polarized laser beam is transformed into circular polarization using a quarter-wave plate. Then, it is incident onto the cascaded telescope composed of POEs through the reflective mirror. By rotating the reflecting mirror, the incident angle can be altered. Figure 2e and f illustrate the steering efficiency and the output angle at different incident angles, respectively. By fitting the output angle and incident angle relationship with the linear function, the line yields a slope of 1.95, which agrees well with the designed magnification factor of 2. Furthermore, the steering efficiency stays above 80 % at a wide angle.

### 2.3 Experimental results

**Figure 3:**
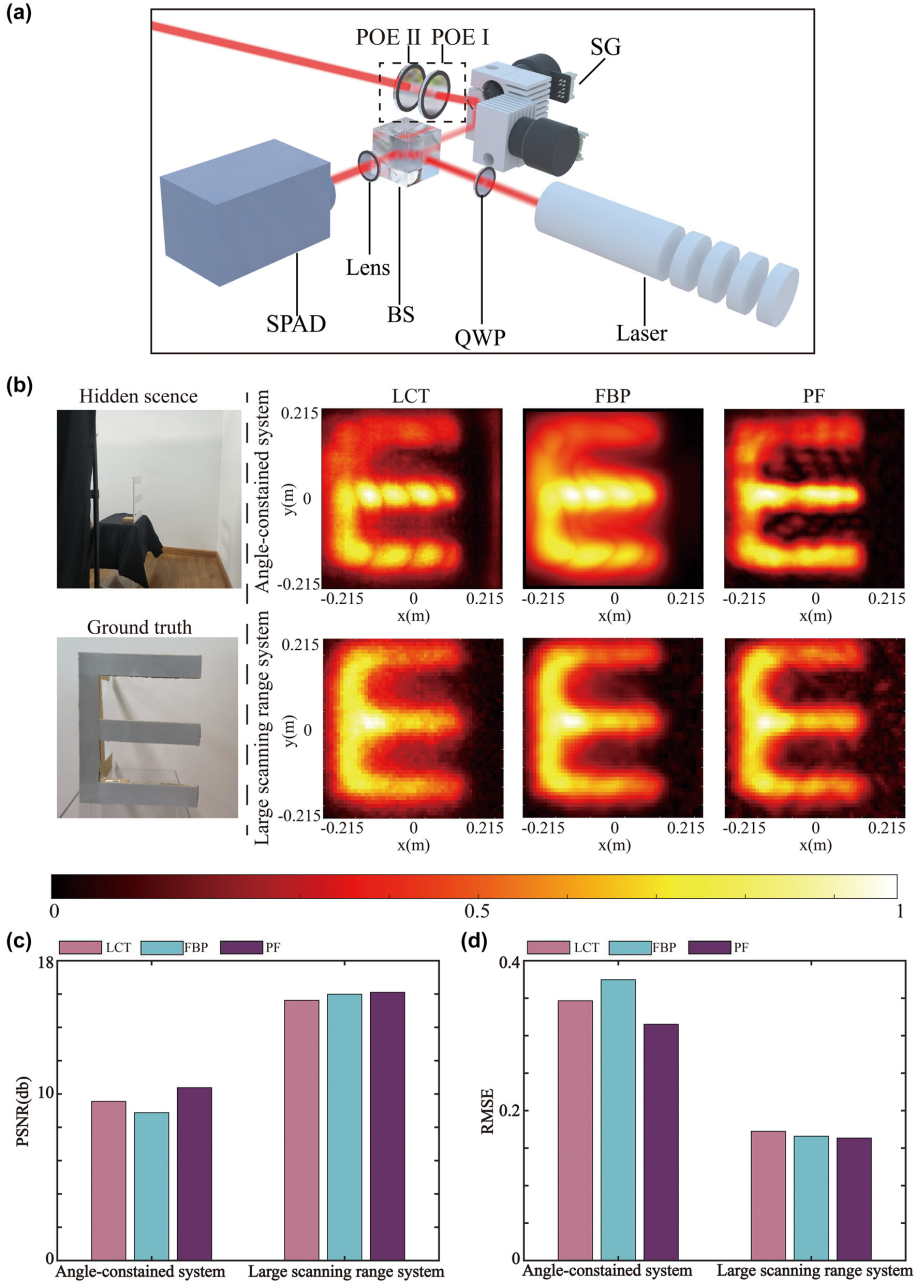
Reconstruction results of the letter “E”. (a) Experimental setup of large scanning range system. SG: scanning galvanometer, BS: beam splitter, QWP: quarter-wave plate, SPAD: single-photon avalanche detector. (b–d) Experimental results (b) the PSNR (c) and (d) the RMSE of the angle-constrained system and the large scanning range system under different reconstruction algorithms.

The detection system is specifically depicted in Figure 3a, where the pulsed laser passes through a beam splitter and a galvanometer for scanning on the relay wall. A cascaded POE forms an angle magnification, enlarging the deflection angle of the beam from the galvanometer. Our system employing POEs is referred to as a large scanning range system. The system without POEs is referred to as an angle-constrained system. After several times of reflection, scattered light from the relay wall passes through the POEs, scanning galvanometer and beam splitter. Finally, they are collected by the single-photon avalanche diode (SPAD). Because the LC-POE elements operate in the geometric phase principle, a quarter-wave plate is added between the laser and the beam splitter to convert linearly polarized light into circularly polarized light.

We demonstrate the effectiveness of the proposed method with experiments on the same object using the angle-constrained NLOS system and our large scanning range system, respectively. A 532 nm pulsed laser with a 24 MHz repetition rate and an average power of 60 mW is used as a light source. After multiple scatterings, the scattered light is recorded by a SPAD with a time jitter of 60 ps. The detection signal is subsequently recorded by a time-correlated single photon counting (TCSPC) module with a time resolution of 4 ps. It is worth noting that both the angle-constrained system and the large scanning range system utilize the same pulse laser, SPAD, and TCSPC. Afterward, we reconstruct the images from the recorded signals collected by the two systems using the light-cone transform (LCT) [1], filtered backprojection (FBP) [2], and phasor field (PF) [7] algorithms, respectively. We then compare the results obtained from each algorithm for both sets of data.

For confocal experiments, we choose the letter “E” to test our method. The measured data are captured at 101 × 101 focal points over a square region of 0.86 × 0.86 m^2^ in the large scanning range system. The hidden scenes are 0.5 m from the illumination wall. To establish a control group under the same experimental conditions, we also capture another set of measurement data using the angle-constrained NLOS system, where the scanning square region is 0.43 × 0.43 m^2^ due to the absence of POEs. Throughout all experiments, the exposure time is 17 min.

The reconstructed results of the letter “E” are displayed in Figure 3b. The maximum intensity projections along the depth direction are represented using the hot colormap. Due to the non-uniformity of the coordinate systems between the reconstructed images obtained from the two systems, we purposely magnify the results from the large scanning range system for easier comparison. In the angle-constrained NLOS imaging system, the upper part of the letter “E” fails to be reconstructed due to the lack of measurement data. In the large scanning range system, the letter “E” can be fully reconstructed, and the results show a significant improvement as compared to those from the angle-constrained system. Regarding the quantitative evaluation, the PSNR of our reconstructed results for the letter “E” are 15.6219 dB, 15.9858 dB, and 16.1009 dB under the LCT, FBP, and PF algorithms, respectively. These values are significantly higher than those obtained by the angle-constrained NLOS system using LCT (9.5535 dB), FBP (8.8779 dB), and PF (10.3791 dB) algorithms. The RMSE of our reconstruction results for the letter “E” are 01724, 0.1659, and 0.1634 under the LCT, FBP, and PF algorithms, respectively. These values are notably smaller than those obtained by the angle-constrained NLOS system using LCT (0.3466), FBP (0.3747), and PF (0.3152) algorithms. The evaluation metrics for the letter “E” are presented in the bar charts. From the bar charts of PSNR and RMSE evaluation metrics in Figure 3c and d, it is evident that the reconstruction results of our system are superior to those of the angle-constrained system. Furthermore, we performed NLOS imaging on more complex three-dimensional objects (see Supplementary Material, Figure S3).

### 2.4 Sparse scanning based on spatial correlation

**Figure 4:**
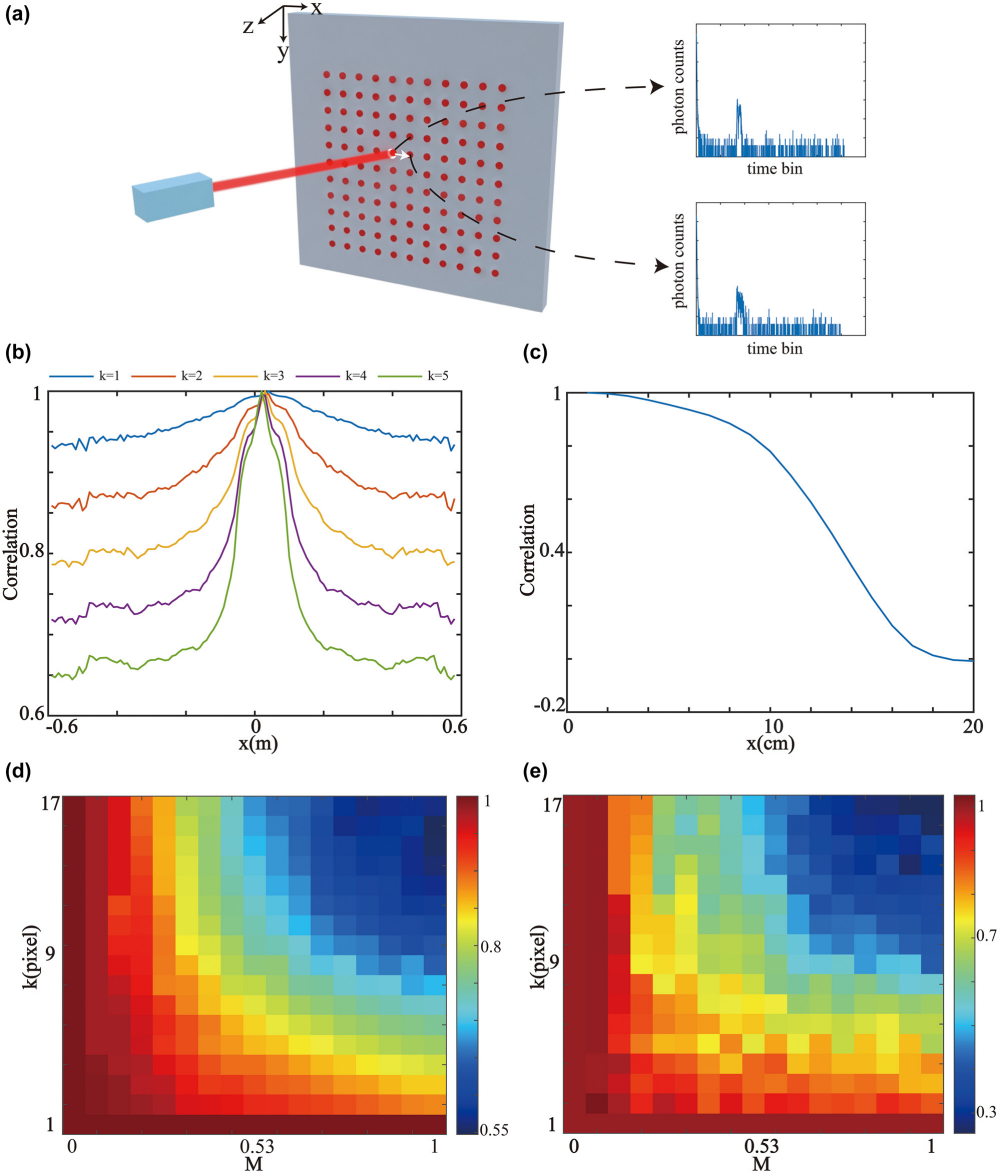
Sparse scanning principle. (a) Measurement data for scanning adjacent points. (b) Correlation function curve at *y* = 0. The five curves represent different intervals. (c) Correlation value of different intervals. (d) PSNR result. (e) SSIM result. *M* represents the ratio of the sparse scanning area width to the overall scanning area width, and *k* represents the sparse scanning interval.

To achieve high-resolution reconstruction, sufficient scanning points over an expanding area are necessary, which greatly impacts the data collection time for NLOS imaging. To overcome this problem, we employ the sparse scanning method illustrated in Figure 1c. This scanning method is based on the correlation of measured data between adjacent sampling points at the same interval on the relay wall. In the Cartesian coordinate system, the position of the laser scanning on the relay wall is given by (*x*, *y*, 0), and the coordinates of the hidden object are represented as (*x*_1_, *y*_1_, *z*_1_). The measured signal is given by:

(8) $$\begin{array}{ll} \varphi \left(x,y,t\right) & =\underset{\Omega }{∭}\frac{1}{r^{4}}g\left(x_{1},y_{1},z_{1}\right)\delta \\ & \;\times \left(2\sqrt{{\left(x-x_{1}\right)}^{2}+{\left(y-y_{1}\right)}^{2}+{z_{1}}^{2}}-tc\right)\\ & \;\times \text{d}x_{1}\text{d}y_{1}\text{d}z_{1} \end{array}$$

where *c* is the speed of light, *δ* is the albedo of the hidden object at point (*x*_1_, *y*_1_, *z*_1_) in the 3D half-space Ω, and *r* is the distance function $r=\sqrt{{\left(x-x_{1}\right)}^{2}+{\left(y-y_{1}\right)}^{2}+{z_{1}}^{2}}$ . The obtained data from two adjacent points, as shown in Figure 4a, can be represented as *φ*(*x*, *y*, *t*) and *φ*(*x* + Δ*x*, *y*, *t*). The Pearson correlation function *d*(*x*) representing the correlation between adjacent samples is

(9) $$\begin{array}{c} d\left(x\right)=\frac{\underset{t}{\sum }\left(\varphi \left(x,y,t\right)-\bar{\varphi \left(x,y,t\right)}\right)\left(\varphi \left(x+\Delta x,y,t\right)-\bar{\varphi \left(x+\Delta x,y,t\right)}\right)}{\sqrt{\underset{t}{\sum }{\left(\varphi \left(x,y,t\right)-\bar{\varphi \left(x,y,t\right)}\right)}^{2}\underset{t}{\sum }{\left(\varphi \left(x+\Delta x,y,t\right)-\bar{\varphi \left(x+\Delta x,y,t\right)}\right)}^{2}}} \end{array}$$

where $\bar{\varphi \left(x,y,t\right)}=\frac{\sum_{t}\varphi \left(x,y,t\right)}{N}$ is the average value, and *N* is the number of time bins. Due to the complexity of Equation (9), we simulate the results using MATLAB software. In the simulation, a letter “E” with dimensions of 0.3 m × 0.3 m is placed at a distance of 0.5 m in front of a 1.2 m × 1.2 m relay surface. The measured data are obtained using a 128 × 128 grid scanning on the relay surface in a confocal NLOS system. Figure 4b shows the correlation function values of the measurements for adjacent samples compared along the *x*-axis. Multiple curves represent measurements taken at different sampling intervals. As the sampling interval increases, the overall correlation of the measurements decreases. However, the spatial correlation coefficients in the middle portion of all curves are relatively high. It implies that reducing the sampling in the middle portion has the least impact on the result. We use the measurements scanned at the central position as a reference to discuss the impact of sampling intervals on the correlation coefficients of measurement data. We show the evolution of the correlation coefficient on the sampling intervals in Figure 4c. The scanning interval occupying ∼7 % of the overall scanning area’s side length can be used for sparse scanning while maintaining the spatial correlation of over 80 %.

Currently, mainstream reconstruction algorithms cannot directly compute the data obtained from sparse scanning and further data processing is required. The primary approach for data processing involves transforming the sparse measurement data into dense grid measurement data using a method like bicubic interpolation. After interpolation, the measurement data from sparse scanning can be transformed into dense grid scanning measurement data. In addition, we also conduct a detailed discussion on the sparse region area and the sparse scanning interval. Figure 4d and e represent the PSNR and the Structure Similarity Index Measure (SSIM) of the sparse scanning results compared to the uniform grid scanning results. The image reconstruction quality decreases as the sparse scanning area and sparse scanning interval increase. However, we consider the sparse scanning to yield satisfactory results when the sparse scanning area is less than 25 % and the scanning interval is less than 6 pixels.

Then, we adopt the sparse scanning approach in our large scanning range system. In this scanning, the total scanning area is 0.86 × 0.86 m^2^ while 0.43 × 0.43 m^2^ in the center is scanned using larger sampling intervals. Suppose we define the ratio of the intermediate sampling interval to the edge sampling interval as a coefficient *k*. As *k* is related to the scanning time, in our experiment with an edge scanning sampling interval of 0.86 cm, the results of different values of *k*, i.e. 1, 2, and 4 are investigated.

**Figure 5:**
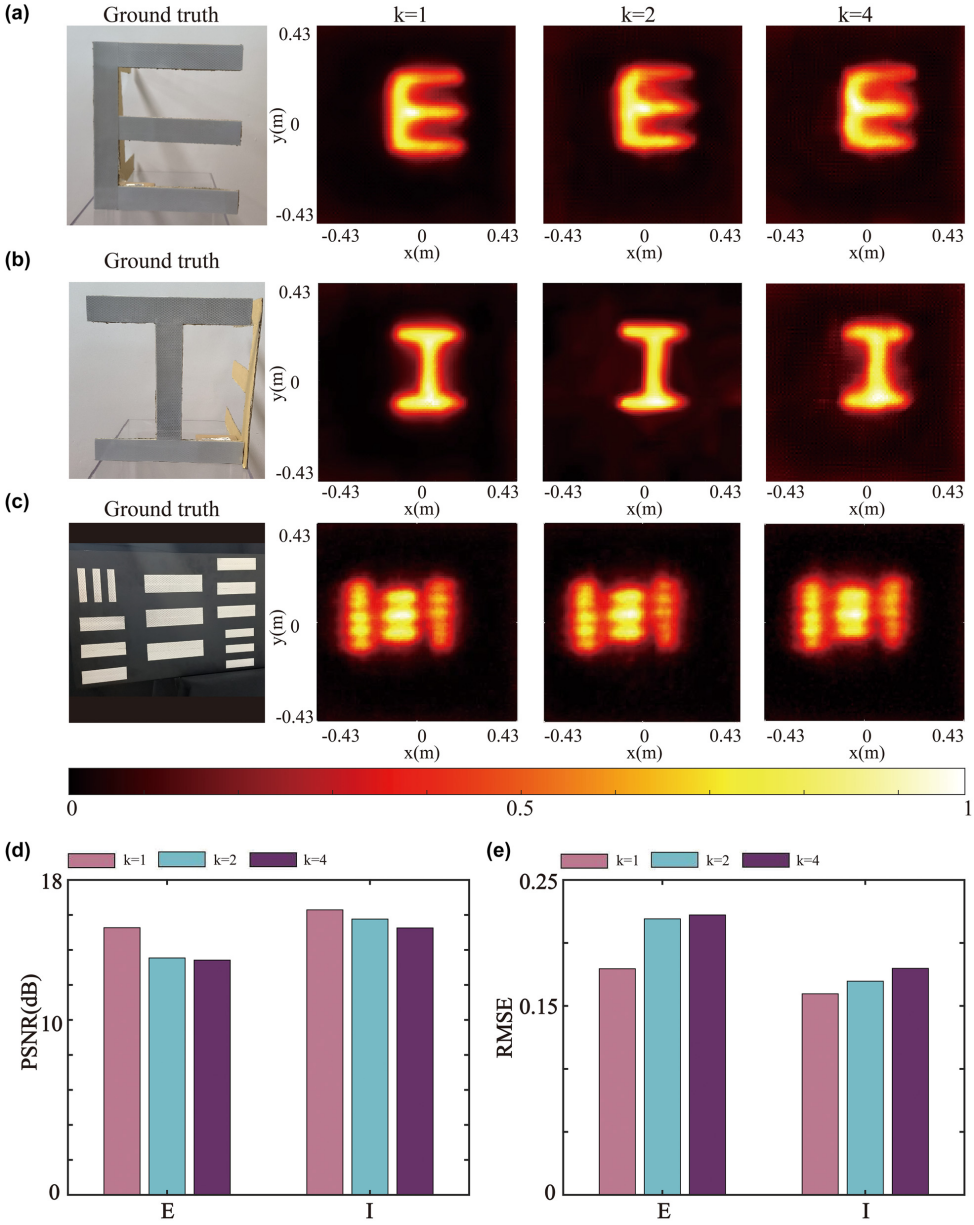
Reconstructed measurement data under sparse scanning. (a–c) Reconstruction results of the letter “E” (a), the letter I (b), and the resolution chart (c). (d–e) PSNR (d) and RMSE (e) for different values of *k*.

Figure 5a–c depicts the reconstruction results under sparse scanning using the LCT algorithm for all outcomes. We also provide the reconstruction results for the FBP and PF algorithm in the Supplementary Material Note S4. Visually, as the central scanning interval increases, there is an increase in reconstruction noise. However, the letter “E” and letter “I” can still be fully reconstructed using the LCT. Consequently, we achieve significant savings in scanning time while maintaining the sampling rate by slightly compromising the image quality. When the scanning coefficient *k* is 2 and 4, it can save 185 s and 234.4 s, respectively. To eliminate background noise, we apply a block-matching and 3D filtering denoising process for the image reconstruction. In Figure 5a–c, it is evident that increasing the value of *k* leads to minor differences in specific details, while significantly reducing the scanning time. Figure 5c displays the results of experiments on the resolution chart. The results indicate a decrease in the reconstruction resolution with the increase of the value of *k*. We can easily find that when the coefficient *k* is 2, the best resolution of the reconstructed image is 3 cm, consistent with the uniform scanning. However, when the coefficient *k* is increased to 4, in the reconstruction results, it is barely possible to distinguish the resolution bars of 4 cm. Moreover, the PSNR and RMSE are used to quantitatively evaluate the reconstruction results under different sparse scanning intervals, as shown in Figure 5d and e. As the sampling interval increases, the evaluation metrics decrease but always remain within an acceptable range.

## 3 Conclusions

In summary, we propose a method that combines sparse scanning with an expanding scanning area to achieve fast, high-resolution NLOS imaging. To this end, we introduce an efficient, compact angle magnification device made of LC-POEs into the NLOS imaging system. Experimental results demonstrate that our designed NLOS system can enhance the image quality and imaging resolution of NLOS imaging. Through sparse scanning, we effectively shorten the data acquisition time by reducing the sampling points in the area with high correlation. We believe that combining sparse scanning with an expanding scanning area can bring a novel perspective to the development of NLOS imaging, extending beyond the limitations of improving reconstruction algorithms and hardware performance. Finally, our method also introduces new insight into real-time NLOS imaging.

## Supplementary Material

Supplementary Material Details

## References

[1] O’Toole M., Lindell D. B., Wetzstein G. (2018). Confocal non-line-of-sight imaging based on the light-cone transform. *Nature*.

[2] Laurenzis M., Velten A. (2014). Nonline-of-sight laser gated viewing of scattered photons. *Opt. Eng.*.

[3] Lindell D. B., Wetzstein G., O’Toole M. (2019). Wave-based non-line-of-sight imaging using fast fk migration. *ACM Trans. Graphics*.

[4] Kirmani A., Hutchison T., Davis J., Raskar R. (2009). Looking around the corner using transient imaging. *2009 IEEE 12th International Conference on Computer Vision*.

[5] Guo C. (2019). Imaging through scattering layers exceeding memory effect range by exploiting prior information. *Opt. Commun.*.

[6] Nam J. H. (2021). Low-latency time-of-flight non-line-of-sight imaging at 5 frames per second. *Nat. Commun.*.

[7] Liu X. (2019). Non-line-of-sight imaging using phasor-field virtual wave optics. *Nature*.

[8] Liu X., Bauer S., Velten A. (2020). Phasor field diffraction based reconstruction for fast non-line-of-sight imaging systems. *Nat. Commun.*.

[9] Xin S., Nousias S., Kutulakos K. N., Sankaranarayanan A. C., Narasimhan S. G., Gkioulekas I. (2019). A theory of Fermat paths for non-line-of-sight shape reconstruction. *Proceedings of the IEEE/CVF Conference on Computer Vision and Pattern Recognition*.

[10] Gariepy G., Tonolini F., Henderson R., Leach J., Faccio D. (2016). Detection and tracking of moving objects hidden from view. *Nat. Photonics*.

[11] Liu X., Wang J., Li Z., Shi Z., Fu X., Qiu L. (2021). Non-line-of-sight reconstruction with signal–object collaborative regularization. *Light Sci. Appl.*.

[12] Liu X., Wang J., Xiao L., Shi Z., Fu X., Qiu L. (2023). Non-line-of-sight imaging with arbitrary illumination and detection pattern. *Nat. Commun.*.

[j_nanoph-2023-0655_ref_013] Xin L., Liangyu H., Yixuan T. (2019). Direct object recognition without line-of-sight using optical coherence. *Proceedings of the IEEE/CVF Conference on Computer Vision and Pattern Recognition*.

[j_nanoph-2023-0655_ref_014] Willomitzer F., Li F., Rangarajan P., Cossairt O. (2018). Non-line-of-sight imaging using superheterodyne interferometry. *Computational Optical Sensing and Imaging*.

[j_nanoph-2023-0655_ref_015] Viswanath A., Rangarajan P., MacFarlane D., Christensen M. P. (2018). Indirect imaging using correlography. *Computational Optical Sensing and Imaging*.

[j_nanoph-2023-0655_ref_016] Smith B. M., O’Toole M., Gupta M. (2018). Tracking multiple objects outside the line of sight using speckle imaging. *Proceedings of the IEEE Conference on Computer Vision and Pattern Recognition*.

[j_nanoph-2023-0655_ref_017] Katz O., Small E., Silberberg Y. (2012). Looking around corners and through thin turbid layers in real time with scattered incoherent light. *Nat. Photon.*.

[18] Boger-Lombard J., Katz O. (2019). Passive optical time-of-flight for non line-of-sight localization. *Nat. Commun.*.

[19] Torralba A., Freeman W. T. (2014). Accidental pinhole and pinspeck cameras: revealing the scene outside the picture. *Int. J. Comput. Vis.*.

[20] Bouman K. L. (2017). Turning corners into cameras: principles and methods. *Proceedings of the IEEE International Conference on Computer Vision*.

[21] Saunders C., Murray-Bruce J., Goyal V. K. (2019). Computational periscopy with an ordinary digital camera. *Nature*.

[22] Katz O., Heidmann P., Fink M., Gigan S. (2014). Non-invasive single-shot imaging through scattering layers and around corners via speckle correlations. *Nat. Photon.*.

[23] Bertolotti J., Van Putten E. G., Blum C., Lagendijk A., Vos W. L., Mosk A. P. (2012). Non-invasive imaging through opaque scattering layers. *Nature*.

[24] Cao R., de Goumoens F., Blochet B., Xu J., Yang C. (2022). High-resolution non-line-of-sight imaging employing active focusing. *Nat. Photonics*.

[25] Zheng S., Liao M., Wang F., He W., Peng X., Situ G. (2021). Non-line-of-sight imaging under white-light illumination: a two-step deep learning approach. *Opt. Express*.

[26] He J., Wu S., Wei R., Zhang Y. (2022). Non-line-of-sight imaging and tracking of moving objects based on deep learning. *Opt. Express*.

[27] Feng X., Gao L. (2021). Ultrafast light field tomography for snapshot transient and non-line-of-sight imaging. *Nat. Commun.*.

[j_nanoph-2023-0655_ref_028] Zhang C. (2020). Low-loss metasurface optics down to the deep ultraviolet region. *Light Sci.*.

[j_nanoph-2023-0655_ref_029] Deng Z.-L. (2018). Diatomic metasurface for vectorial holography. *Nano Lett.*.

[j_nanoph-2023-0655_ref_030] Tang D. (2015). Ultrabroadband superoscillatory lens composed by plasmonic metasurfaces for subdiffraction light focusing. *Laser Photon. Rev.*.

[j_nanoph-2023-0655_ref_031] Qin F. (2016). Hybrid bilayer plasmonic metasurface efficiently manipulates visible light. *Sci. Adv.*.

[32] Yang Y., Kravchenko I. I., Briggs D. P., Valentine J. (2014). All-dielectric metasurface analogue of electromagnetically induced transparency. *Nat. Commun.*.

[j_nanoph-2023-0655_ref_033] Li G. (2015). Continuous control of the nonlinearity phase for harmonic generations. *Nat. Mater.*.

[j_nanoph-2023-0655_ref_034] Li J. (2021). From lingering to rift: metasurface decoupling for near-and far-field functionalization. *Adv. Mater.*.

[j_nanoph-2023-0655_ref_035] Fan Q. (2022). Trilobite-inspired neural nanophotonic light-field camera with extreme depth-of-field. *Nat. Commun.*.

[j_nanoph-2023-0655_ref_036] Fei Z., Yinghui G., Mingbo P., Xiong L., Xiaoliang M., Xiangang L. (2020). Metasurfaces enabled by asymmetric photonic spin-orbit interactions. *Opto-Electron. Eng.*.

[j_nanoph-2023-0655_ref_037] Fan Z.-B. (2018). Silicon nitride metalenses for close-to-one numerical aperture and wide-angle visible imaging. *Phys. Rev. Appl.*.

[j_nanoph-2023-0655_ref_038] Zhang F. (2023). Meta-optics empowered vector visual cryptography for high security and rapid decryption. *Nat. Commun.*.

[j_nanoph-2023-0655_ref_039] Zhang R. (2023). Angular superoscillatory metalens empowers single-shot measurement of OAM modes with finer intervals. *Adv. Opt. Mater.*.

[j_nanoph-2023-0655_ref_040] Xie X. (2021). Generalized Pancharatnam–Berry phase in rotationally symmetric meta-atoms. *Phys. Rev. Lett.*.

[41] Pu M. (2015). Catenary optics for achromatic generation of perfect optical angular momentum. *Sci. Adv.*.

[42] Guo Y. (2021). Spin-decoupled metasurface for simultaneous detection of spin and orbital angular momenta via momentum transformation. *Light Sci. Appl.*.

[43] Zhang F. (2017). All‐dielectric metasurfaces for simultaneous giant circular asymmetric transmission and wavefront shaping based on asymmetric photonic spin–orbit interactions. *Adv. Funct. Mater.*.

[44] Deng Z. L., Li F. J., Li H., Li X., Alù A. (2022). Extreme diffraction control in metagratings leveraging bound states in the continuum and exceptional points. *Laser Photon. Rev.*.

[45] Feng Z. (2023). Dual-band polarized upconversion photoluminescence enhanced by resonant dielectric metasurfaces. ..

[j_nanoph-2023-0655_ref_046] Kobashi J., Yoshida H., Ozaki M. (2016). Planar optics with patterned chiral liquid crystals. *Nat. Photonics*.

[j_nanoph-2023-0655_ref_047] Chen J. (2022). Planar wide-angle-imaging camera enabled by metalens array. *Optica*.

[j_nanoph-2023-0655_ref_048] Ye X. (2022). Chip-scale metalens microscope for wide-field and depth-of-field imaging. *Adv. Photon.*.

[j_nanoph-2023-0655_ref_049] Lei Y. (2023). Snapshot multi-dimensional computational imaging through a liquid crystal diffuser. *Photon. Res.*.

[j_nanoph-2023-0655_ref_050] Tang D. (2022). Simultaneous surface display and holography enabled by flat liquid crystal elements. *Laser Photon. Rev.*.

[j_nanoph-2023-0655_ref_051] Xu Q., Sun T., Wang C. (2021). Coded liquid crystal metasurface for achromatic imaging in the broadband wavelength range. *ACS Appl. Nano Mater.*.

[j_nanoph-2023-0655_ref_052] Lu X. (2022). Broadband high-efficiency polymerized liquid crystal metasurfaces with spin-multiplexed functionalities in the visible. *Photon. Res.*.

[j_nanoph-2023-0655_ref_053] Ji Y. (2021). Active terahertz spin state and optical chirality in liquid crystal chiral metasurface. *Phys. Rev. Mater.*.

[j_nanoph-2023-0655_ref_054] Zhou S., Shen Z., Li X., Ge S., Lu Y., Hu W. (2020). Liquid crystal integrated metalens with dynamic focusing property. *Opt. Lett.*.

[55] Zhang F. (2021). Extreme-angle silicon infrared optics enabled by streamlined surfaces. *Adv. Mater.*.

[56] Si G., Zhao Y., Leong E. S. P., Liu Y. J. (2014). Liquid-crystal-enabled active plasmonics: a review. *Materials*.

[57] Lee K. M., Tondiglia V. P., White T. J. (2018). Electrically reconfigurable liquid crystalline mirrors. *ACS Omega*.

[58] Albero J. (2012). Liquid crystal devices for the reconfigurable generation of optical vortices. *J. Lightwave Technol.*.

[59] Jiang D. (2019). Liquid crystal-based wideband reconfigurable leaky wave X-band antenna. *IEEE Access*.

[60] Zhan T. (2019). Pancharatnam–Berry optical elements for head-up and near-eye displays. *JOSA B*.

[61] He Z., Yin K., Wu S.-T. (2021). Applications Miniature planar telescopes for efficient, wide-angle, high-precision beam steering. *Light Sci.*.

[62] Tachella J. (2019). Bayesian 3D reconstruction of complex scenes from single-photon lidar data. *SIAM J. Imag. Sci.*.

[63] Halimi A., Tobin R., McCarthy A., Bioucas-Dias J., McLaughlin S., Buller G. S. (2019). Robust restoration of sparse multidimensional single-photon LiDAR images. *IEEE Trans. Comput. Imag.*.

