# Supplementary material for: High-resolution non-line-of-sight imaging based on liquid crystal planar optical elements
Source: Nanophotonics. 2024 Jan 10;13(12):2161–72. doi: 10.1515/nanoph-2023-0655 (PMC11501925; doi:10.1515/nanoph-2023-0655)
Supplement: Supplementary file 1 — Supplementary Material Details [file j_nanoph-2023-0655_suppl_001.docx]

Short title: Planar non-line-of-sight imaging

Supplementary Material

**High-resolution non-line-of-sight imaging based on liquid crystal planar optical elements**

*Zhibin Zhao#, Qi Zhang#, Xiaoyin Li, Yinghui Guo, Mingbo Pu, Fei Zhang, Hengshuo Guo, Zewei Wang, Yulong Fan, Mingfeng Xu, Xiangang Luo**

Z. Zhao, Q. Zhang, X. Li, Y. Guo, M. Pu, F. Zhang, Z. Wang, F. Yu, M. Xu, X. Luo

National Key Laboratory of Optical Filed Manipulation Science and Technology, Institute of Optics and Electronics, Chinese Academy of Sciences, Chengdu 610209, China

State Key Laboratory of Optical Technologies on Nano-Fabrication and Micro-Engineering, Institute of Optics and Electronics, Chinese Academy of Sciences, Chengdu 610209, China

Z. Zhao and Q. Zhang contributed equally to this work.

E-mail: lxg@ioe.ac.cn

Z. Zhao, Q. Zhang, X. Li, Y. Guo, M. Pu, F. Zhang, Z. Wang, M. Xu.

Research Center on Vector Optical Fields, Institute of Optics and Electronics, Chinese Academy of Sciences, Chengdu 610209, China.

Z. Zhao, Z. Wang, M. Pu, Y. Guo, X. Luo

School of Optoelectronics, University of Chinese Academy of Sciences, Beijing 100049, China.

H. Guo

Tianfu Xinglong Lake Laboratory, Chengdu 610299, China

**Note S1. Lateral resolution limits**

The lateral resolution of NLOS imaging is primarily constrained by the scanning area 2*w*×2*w*, the distance between hidden objects *z* and the wall, and the system jitter $\Delta t$ [1]. In non-confocal NLOS imaging scenarios, the illumination point *p* and the detection point *r* do not have spatial overlap, and $q_{1}$ and $q_{2}$ represent two scatterers with a lateral spacing of *Δd* (Figure S1). Specifically, two scattering points, $q_{1}$ and $q_{2}$, are resolvable in space only if their indirect signals are resolvable in time:

$abs\left( \left( \left\| p-q_{1} \right\|_{2}+\left\| r-q_{1} \right\|_{2} \right)-\left( \left\| p-q_{2} \right\|_{2}+\left\| r-q_{2} \right\|_{2} \right) \right)\geq c\Delta t$ (S1)

Equation (S1) can be rewritten as:

$abs(\left( \left\| p-q_{1} \right\|_{2}-\left\| p-q_{2} \right\|_{2} \right)+(\left\| r-q_{1} \right\|_{2}-\left\| r-q_{2} \right\|_{2}))\geq c\Delta t$ (S2)

We assume that *Δd* is much smaller than the wall width *w*, such that $\theta_{1}\approx\theta_{2}$. According to geometric relationships, $abs\left( \left\| p-q_{1} \right\|_{2}-\left\| p-q_{2} \right\|_{2} \right)$ can be expressed as:

$\Delta dcos\left( \theta_{1} \right)\approx\Delta dcos\left( \theta_{2} \right)=\Delta dcos\left( {tan}^{-1}\left( \frac{z}{w} \right) \right)=\Delta d\frac{w}{\sqrt{w^{2}+z^{2}}}$ (S3)

Then,

$\Delta d\frac{w}{\sqrt{w^{2}+z^{2}}}+\delta x\geq c\Delta t$ (S4)

where δx is the path difference of the hidden object from the detection point on the wall. The minimum lateral distance between two points is

$\Delta d\geq\frac{\sqrt{w^{2}+z^{2}}\left( c\Delta t-\delta x \right)}{w}$ (S5)

In a non-confocal system, the detector is typically aimed at the center position of the wall, so that δx takes a very small value. In the confocal system, where the detection point and the illumination point coincide, the $\delta x$ is equal to $\Delta d\frac{w}{\sqrt{w^{2}+z^{2}}}$. The minimum lateral distance can be expressed as:

$\Delta d\geq\frac{\sqrt{w^{2}+z^{2}}}{2w}$ (S6)


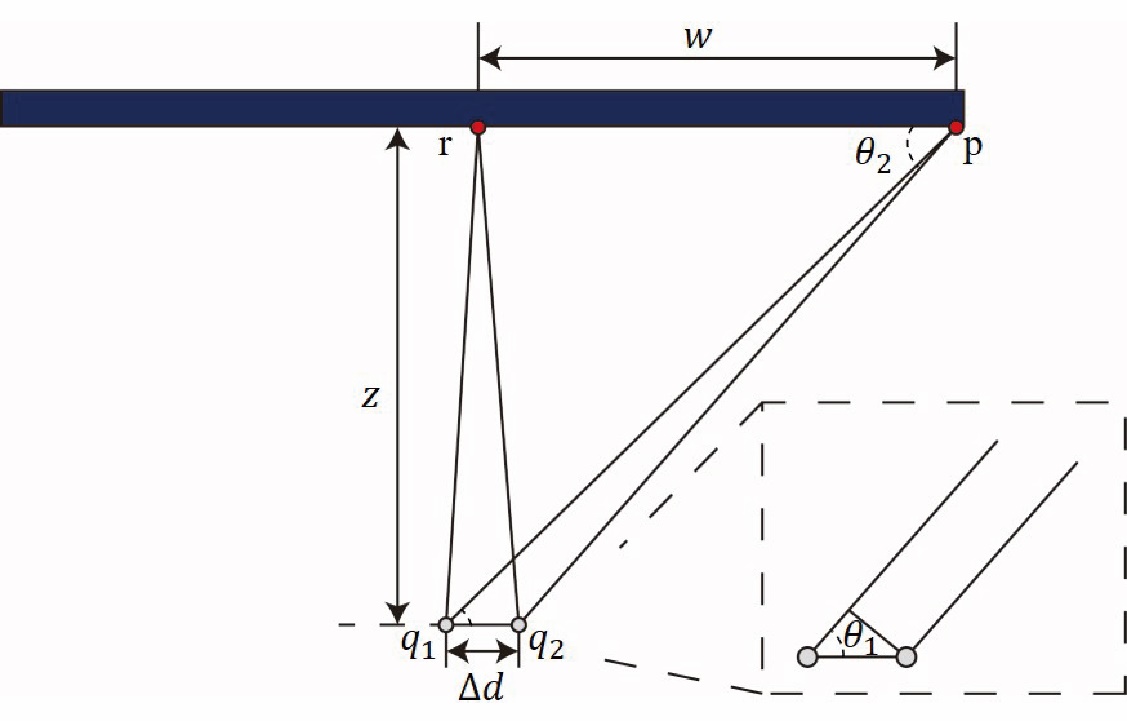


**Figure S1.** Illustration of the lateral resolution.

**Note S2. Simulation results**

According to Equation (3), the sampling interval also affects the image resolution of the reconstruction. We verify this conclusion through simulation. The hidden object is a custom resolution chart with resolution bars spaced at distances of 1cm, 2cm, 3cm, and 4cm, as shown in Figure S2a. The hidden object is located 0.5m in front of the wall. The scanning area is 0.6m×0.6m. In the confocal mode, we generate NLOS imaging data for three groups with different scanning point numbers. Figures 2Sb-d depict the reconstruction results of these three groups using the LCT algorithm. Figures Sb-d represent oversampling, proper sampling, and undersampling, respectively. To validate the simulation results, we calculate the maximum scanning interval satisfying the Nyquist sampling rate of 1cm. The sampling intervals for Figures Sb-d are 0.85cm, 1cm, and 2cm, representing the reconstruction results for oversampling, proper sampling, and undersampling, respectively. The simulation results for oversampling and proper sampling can distinguish 2cm resolution bars, but the simulation results for undersampling cannot distinguish 2cm resolution bars. Furthermore, the oversampling results do not show any further improvement in resolution compared to the proper sampling results. This demonstrates the reliability of Equation (3) and the simulation results.


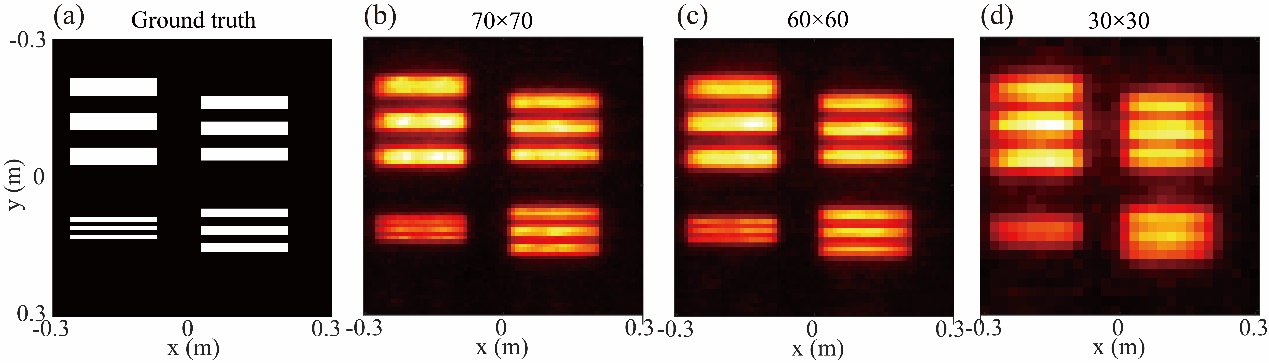


**Figure S2**. Reconstruction of synthetic data. a) The ground truth. b) -d) Reconstruction results with different scanning intervals.

**Note S3 NLOS imaging results of three-dimensional objects**

After setting up the confocal NLOS imaging system as described in the main text, we also perform NLOS imaging on some more complex objects. Figure S3b shows the NLOS results for a swan sculpture and a humanoid gypsum sculpture. The measurement data for the swan sculpture and the gypsum humanoid figure are obtained by scanning a 0.4m×0.4m area on the wall with 64×64 points and scanning a 0.8m×0.8m area on the wall with 128×128 points, respectively. In both sets of experiments, the exposure time for each scanning point is 0.1 seconds. After obtaining the measurement data, we reconstruct the two sets of experimental data using the LCT, FBP, and PF algorithms, respectively. As shown in Figure S3, under different reconstruction algorithms, we can image the swan sculpture and the humanoid gypsum sculpture completely, and distinguish the approximate outlines of the objects. This indicates that our system has the capability to perform NLOS imaging of complex objects.
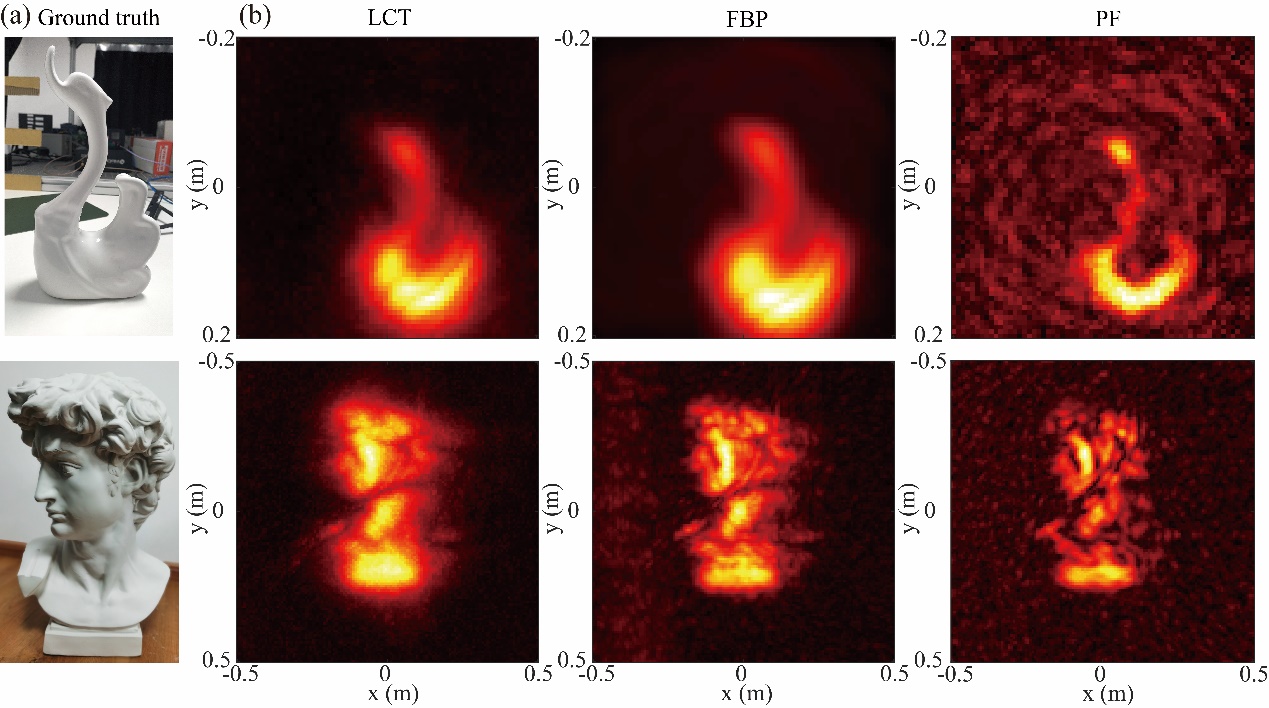


**Figure S3.** Reconstruction of measured data. a) Photographs of the hidden object. b) Experimental results under different algorithms.

**Note S4. Imaging results of sparse scanning under different algorithms**

Figure S4 shows the reconstruction results under sparse scanning using the FBP and PF algorithms. It can be observed that both FBP and PF algorithms are capable of imaging the letters “E” and “I”, demonstrating the robustness of our method. As shown in Figure S4, both the FBP algorithm and the PF algorithm can reconstruct measurement data obtained at different sparse scanning intervals. Both the letter “E” and the letter “I” can be fully reconstructed, and the overall imaging results are satisfactory. Consistent with the conclusions obtained in the main text, as the sparse scanning interval increases, the visual quality of the reconstruction results appears to slightly degrade. However, at the cost of sacrificing some image quality, it is possible to save up to approximately 20% of measurement data acquisition time.


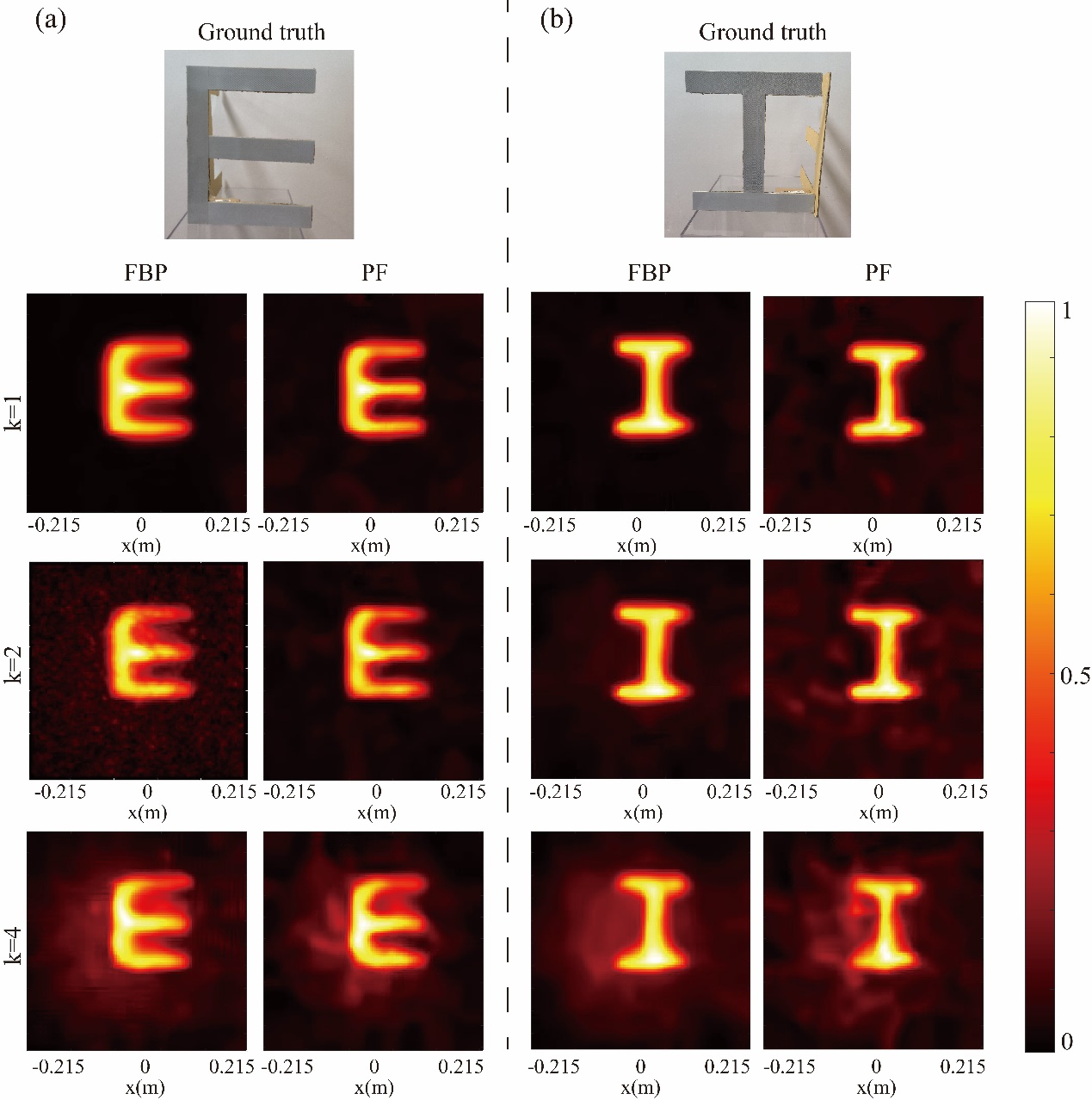


**Figure S4.** Reconstructed measurement data under sparse scanning. a) The reconstruction results of the letter 'E' under the FBP and PF algorithms. b) The reconstruction results of the letter 'I' under the FBP and PF algorithms.

**References**

[1] M. O’Toole, D. B. Lindell, and G. Wetzstein, "Confocal non-line-of-sight imaging based on the light-cone transform," *Nature,* vol. 555, no. 7696, pp. 338-341, 2018.
